# The Role of Txnip in Mediating Low-Magnesium-Driven Endothelial Dysfunction

**DOI:** 10.3390/ijms24098351

**Published:** 2023-05-06

**Authors:** Laura Locatelli, Giorgia Fedele, Jeanette A. Maier

**Affiliations:** Department of Biomedical and Clinical Sciences, Università di Milano, Via GB Grassi 74, 20157 Milano, Italy; laura.locatelli@unimi.it (L.L.); giorgia.fedele@unimi.it (G.F.)

**Keywords:** magnesium, HUVEC, permeability, lipid droplets

## Abstract

Magnesium deficiency is associated with a greater risk of developing cardiovascular diseases since this cation is fundamental in regulating vascular function. This clinical evidence is sustained by in vitro studies showing that culturing endothelial cells in low concentrations of magnesium promotes the acquisition of a pro-oxidant and pro-inflammatory phenotype. Here, we show that the increase in reactive oxygen species in endothelial cells in low-magnesium-containing medium is due to the upregulation of the pro-oxidant protein thioredoxin interacting protein (TXNIP), with a consequent accumulation of lipid droplets and increase in endothelial permeability through the downregulation and relocalization of junctional proteins. Silencing TXNIP restores the endothelial barrier and lipid content. Because (i) mitochondria serve multiple roles in shaping cell function, health and survival and (ii) mitochondria are the main intracellular stores of magnesium, it is of note that no significant alterations were detected in their morphology and dynamics in our experimental model. We conclude that TXNIP upregulation contributes to low-magnesium-induced endothelial dysfunction in vitro.

## 1. Introduction

Vascular endothelial cells are fundamental gatekeepers of cardiovascular health since they regulate vessel tone, blood flow, coagulation, inflammation and smooth muscle cell proliferation [1]. Accordingly, injury or dysfunction of the endothelium leads to different diseases. Magnesium (Mg) deficiency is one of the many factors impairing endothelial function [2,3]. Mg is introduced in the diet through both food and water, and in the human body it represents the fourth most abundant mineral after calcium, potassium and sodium [2]. Intracellularly, Mg acts as a cofactor of hundreds of enzymes, participating in reactions involved in all the metabolic pathways, and it contributes to the regulation of membrane stability, regulates ion channels and serves as an intracellular signal [4,5]. Epidemiological studies have shown that poor Mg intakes are associated with a greater risk of developing cardiovascular diseases (arrhythmia, coronary heart disease, pre-eclampsia, heart failure), neurological diseases (seizures), depression and respiratory diseases [6,7,8]. In vitro, low Mg triggers endothelial dysfunction by promoting the acquisition of a pro-oxidant and pro-inflammatory phenotype, impairing the antioxidant defenses, modulating gene expression, retarding cell growth and promoting senescence [3,9,10]. In a previous work, we showed that human endothelial cells from the umbilical vein (HUVEC), a widely used model for macrovascular endothelial cells, react to 24 h of culture in low Mg with an increase in the levels of Peroxisome Proliferator Activated Receptor gamma (PPAR-γ) and its transcriptional coactivator Endothelial Differentiation-related Factor-1 (EDF-1), which promote the deposition of lipids through a reactive oxygen species (ROS)-dependent mechanism [11]. It is known that endothelial cells can store neutral lipids in lipid droplets [12], cytosolic fat storage organelles that are dynamically synthesized in response to cellular needs or different stimuli [13]. Far from being only an inert reservoir of triglycerides, lipid droplets are emerging as important regulators of different pathways. They regulate the redox status of the cells protecting them from lipotoxicity [14]. In addition, they maintain energy homeostasis, interact with mitochondria and participate in the regulation of the membrane composition. Oxidative stress, nutritional variation and energy imbalance induce their biogenesis [13]. 

A fundamental role of the endothelium is to maintain the barrier function, which is essential for the proper compartmentalization of the vascular and interstitial spaces. Failure to maintain barrier integrity results in the leakage of fluid and proteins from the vasculature into the interstitial space, an event involved in many diseases, particularly conditions such as sepsis that can be fatal [15]. Various pro-atherogenic stimuli increase macrovascular endothelial cell permeability through ROS overproduction and the consequent pro-inflammatory status [16]. This leads to rearrangements of cell-to-cell junctions, which facilitate the subendothelial deposition of low-density lipoproteins. Indeed, the distribution of VE-cadherin, pivotal in maintaining the endothelial barrier, is disorganized in atherosclerosis [17]. Considering that Mg deficiency has a role in atherogenesis [2,3,18,19,20], we recall a previous study demonstrating that Mg deprivation increases endothelial permeability in vitro and in vivo through the S1P1-Rac1 pathways [21]. However, no data are available about junctional proteins at the moment.

Here, we investigated how an acute severe reduction in extracellular Mg (0.1 mM Mg for 24 h) generates oxidative stress in HUVEC. We also analyzed endothelial permeability and lipid accumulation in the same experimental conditions. 

## 2. Results

### 2.1. Culture in Low-Mg Medium Does Not Impact the Intracellular Mg and Calcium Levels

**Figure 1:**
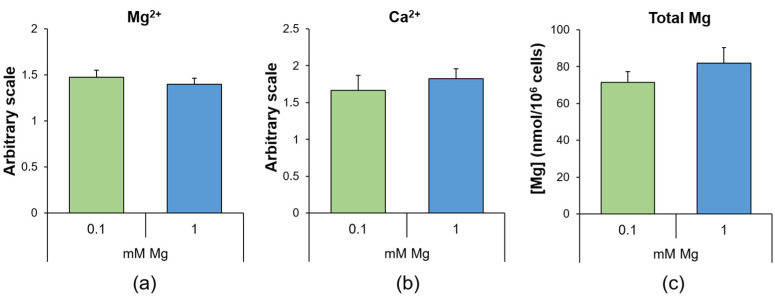
Mg deficiency does not modulate intracellular Ca and Mg levels. In HUVEC cultured for 24 h in 0.1 (low) or 1 (physiological) mM Mg, we measured (**a**) free Mg^2+^ using Mag-fura-2AM, (**b**) free Ca^2+^ using Fura-2AM and (**c**) total Mg using the fluorescent probe DCHQ5. The experiments were performed three times in triplicate, and the data are presented as the mean ± standard deviation (SD).

Human umbilical vein endothelial cells (HUVEC) were cultured in medium containing a normal (1 mM) or low (0.1 mM) concentration of extracellular Mg for 24 h to evaluate if the culture in medium containing 0.1 mM Mg might affect the intracellular concentrations of magnesium and calcium ions (Mg^2+^ and Ca^2+^). We measured free Mg^2+^ and Ca^2+^ using Mag-fura-2AM and Fura-2AM, respectively, and found no differences between HUVEC in 0.1 and 1 mM Mg. In addition, we found that the concentration of total Mg, measured with the fluorescent probe DCHQ5 [22], did not vary under the two experimental conditions (Figure 1).

### 2.2. Culture in Low-Mg Medium Induces Cytosolic ROS Accumulation by Upregulating TXNIP

**Figure 2:**
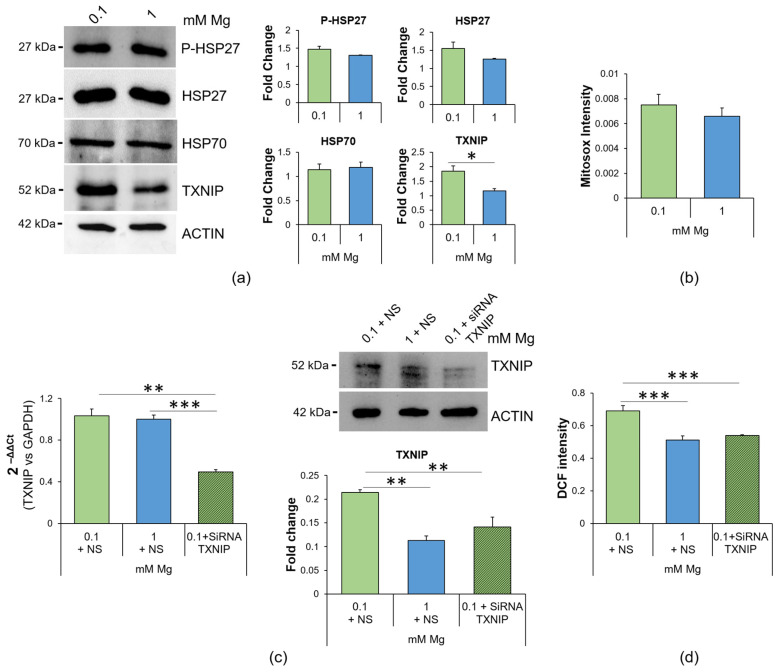
Mg deficiency alters the redox balance in HUVEC, and TXNIP silencing prevents ROS accumulation. (**a**) Western blots were performed on protein lysates of HUVEC cultured for 24 h in 0.1 or 1 mM Mg using antibodies against P-HSP27, HSP27, HSP70 and TXNIP. Anti-β-actin antibodies were used as a control of equal loading. A representative blot of three independent experiments is shown. Densitometry of the bands was performed with the software ImageLab, and the results are expressed as the fold change compared to the 1 mM control ± SD. (**b**) Mitochondrial ROS were analyzed using the fluorescent probe Mitosox. The results are expressed as the mean fluorescence of three independent experiments performed in triplicate ± SD. (**c**) Real-time PCR and Western blotting were performed on HUVEC cultured for 24 h in 0.1 mM Mg + NS (non-silencing siRNA), 1 mM Mg + NS and 0.1 mM Mg + siRNA TXNIP. For the real-time PCR, TaqMan Gene Expression Assay probes for TXNIP and glyceraldehyde-3-phosphate dehydrogenase (GAPDH) were used. The housekeeping gene GAPDH was used as an internal reference gene. Relative changes in gene expression were analyzed using the 2^−ΔΔCt^ method. Western blots were performed using antibodies against TXNIP. Anti-β-actin antibodies were used as a control of equal loading. A representative blot is shown. The experiment was performed in triplicate three times. Data are expressed as means ± SD. (**d**) ROS content was measured using the DCFDA probe (λexc = 495 nm, λemm = 529 nm), and the results are expressed as the mean fluorescence of three independent experiments performed in triplicate ± SD. * *p* ≤ 0.05; ** *p* ≤ 0.01; *** *p* ≤ 0.001.

Since low Mg concentrations are associated with ROS production [11,23,24], we investigated whether the accumulation of ROS was due to an imbalance in the levels of proteins involved in redox homeostasis. Through Western blotting, we analyzed the levels of two stress proteins also implicated in protecting against oxidative stress [25,26], i.e., Heat Shock Protein (HSP)27 and HSP70, and the levels of the pro-oxidant protein TXNIP. We found that low Mg concentration did not affect HSP27, phosphorylated (P-HSP27) or not, or HSP70 levels, while it increased TXNIP (Figure 2a). We then measured mitochondrial ROS using the fluorescent dye Mitosox and found no differences between cells cultured in 0.1 or 1.0 mM Mg (Figure 2b), thus indicating the cytosolic source of ROS. To demonstrate the role of TXNIP upregulation in low-Mg-induced accumulation of ROS, we silenced it using siRNA. qRT-PCR and Western blotting showed successful silencing (Figure 2c). Then, we measured the amount of ROS using the DCFDA probe. Figure 2d shows that silencing TXNIP reduced ROS to control levels.

### 2.3. Culture in Low-Mg Medium Does Not Affect Mitochondria

**Figure 3:**
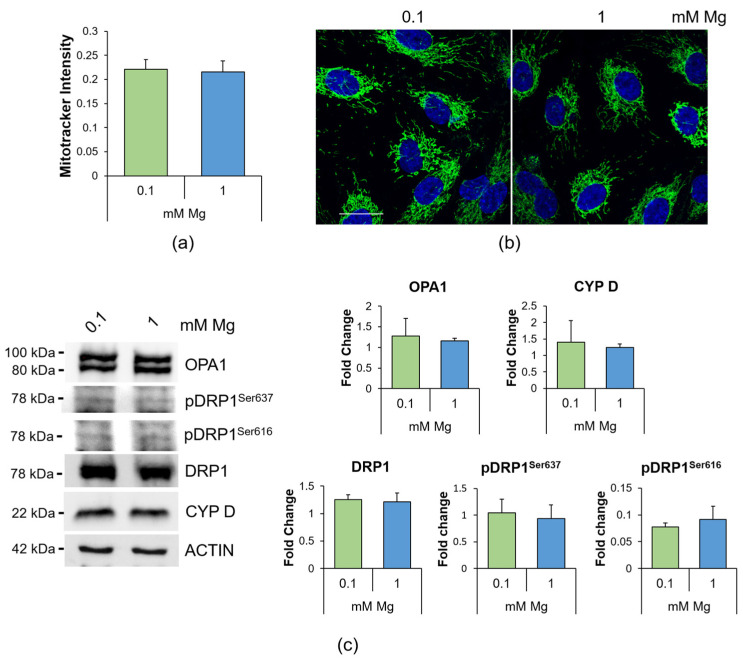
Mitochondrial morphology and dynamics in HUVEC cultured in 0.1 or 1 mM Mg for 24 h. (**a**) Mitochondrial content was measured using the fluorescent probe MitoTracker (λexc = 570 nm, λemm = 599 nm). The results are expressed as the mean fluorescence of three independent experiments performed in triplicate ± SD. (**b**) Immunofluorescence was performed on HUVEC using anti-CYP D antibodies (green). 4′,6-Diamidino-2-phenylindole (DAPI) (blue) was used to stain the nuclei. Images were acquired using a 40× objective in oil with an SP8 Leica confocal microscope. Scale bar: 20 µm. (**c**) Western blots were performed using antibodies against DRP1, pDRP1^Ser616^, pDRP1^Ser637^, OPA1 and CYP D. Anti-β-actin antibodies were used as a control of equal loading. A representative blot of three independent experiments is shown. Densitometry of the bands was performed with the software ImageLab, and the results are expressed as the fold change compared to the 1 mM control ± SD.

Because ROS excess leads to mitochondrial dysfunction [27] and Mg also regulates mitochondrial function [28], we analyzed mitochondria as follows: (i) quantification of the mitochondrial content through MitoTracker (Figure 3a); (ii) detection of the morphology of the organelles via immunofluorescence using anti-cyclophilin D (CYP D) antibodies (Figure 3b); (iii) assessment of mitochondrial dynamics (Figure 3c). We found that the mitochondrial content and morphology were not affected in low-Mg-cultured HUVEC (Figure 3a,b), in agreement with the conservation of the balance between the fission protein dynamin related protein (DRP)1, its phosphorylated forms on Serine 616 and 637 (pDRP1^Ser616^ and pDRP1^Ser637^), which are activatory and inhibitory, respectively, and the fusion protein optic atrophy (OPA)1 (Figure 3c).

### 2.4. Culture in Low-Mg Medium Induces the Accumulation of Lipid Droplets through TXNIP Upregulation

**Figure 4:**
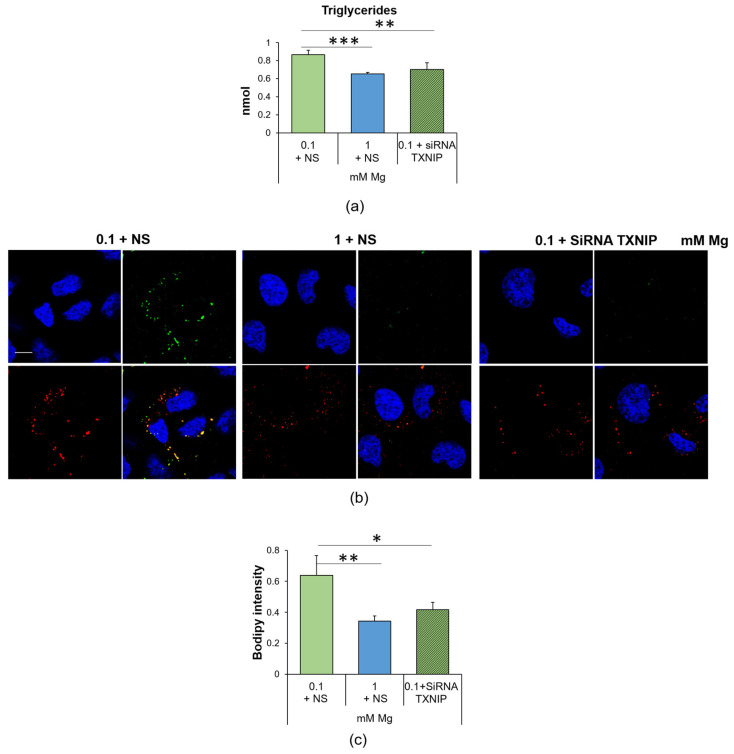
Low Mg medium-induced accumulation of lipid droplets is linked to the upregulation of TXNIP. (**a**) Triglyceride quantification was performed on HUVEC cultured for 24 h in 0.1 mM Mg + NS, 1 mM Mg + NS or 0.1 mM of Mg + siRNA TXNIP. Data are expressed as nmol of triglycerides. The results are the mean of three separate experiments in triplicate ± SD. (**b**) Confocal microscopy was performed on HUVEC using the bodipy probe (green) and antibodies against PLIN-2 (red). The merging of the colors is shown in yellow. DAPI (blue) was used to stain the nuclei. Images were acquired using a 40× objective in oil with an SP8 Leica confocal microscope and zoomed. Scale bar: 10 µm. (**c**) Lipids were measured using the bodipy probe (λexc = 493 nm, λemm = 503 nm), and the results are expressed as the mean fluorescence of three independent experiments performed in triplicate ± SD. * *p* ≤ 0.05; ** *p* ≤ 0.01; *** *p* ≤ 0.001.

In a previous study, we reported that HUVEC cultured in Mg-deficient medium accumulated lipids [11]. To gain more insight into this issue, we initially measured the total amount of triglycerides and found it increased after the cells were cultured in medium containing 0.1 mM Mg for 24 h (Figure 4a). By co-staining the samples with bodipy and the lipid droplet protein Perilipin-2 (PLIN2), we visualized vesicles laden with neutral lipids and surrounded by PLIN2-enriched membranes in low-Mg-cultured HUVEC, thereby demonstrating a marked increase in lipid droplets in Mg-deficient HUVEC (Figure 4b). 

We also found that TXNIP silencing prevented the accumulation of triglycerides and lipid droplets in HUVEC cultured in Mg-deficient medium (Figure 4). Silencing TXNIP in HUVEC in 1 mM Mg exerted no effect [29].

### 2.5. Culture in Low-Mg Medium Increases Endothelial Permeability through TXNIP Upregulation

**Figure 5:**
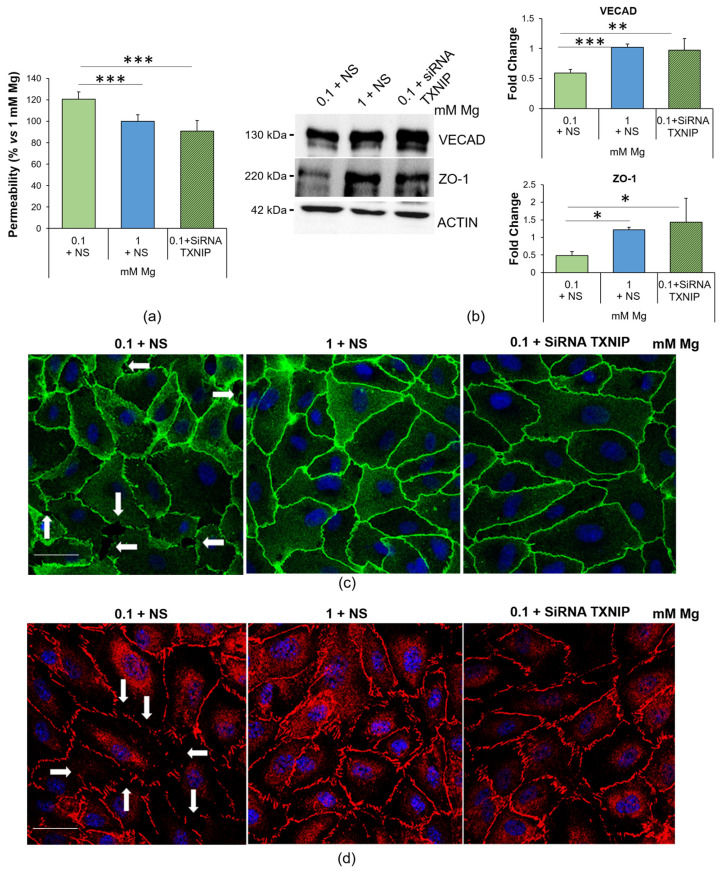
Low-Mg medium increases endothelial permeability through the upregulation of TXNIP. (**a**) Permeability was measured using the transwell method on HUVEC cultured in 0.1 mM Mg + NS, 1 mM Mg + NS or 0.1 + siRNA TXNIP mM Mg for 24 h. The data are expressed as the percentage vs. 1 mM control ± SD. (**b**) Western blots were performed using antibodies against VECAD and ZO-1. Anti-β-actin antibodies were used as a control of equal loading. A representative blot of three independent experiments is shown. Densitometry of the bands was performed with the software ImageLab, and the results are expressed as the fold change compared to the control (1 mM Mg + NS) ± SD. (**c**,**d**) Immunofluorescence was performed on HUVEC using antibodies against VECAD (**c**, green) and ZO-1 (**d**, red). DAPI (blue) was used to stain the nuclei. Images were acquired using a 40× objective in oil with an SP8 Leica confocal microscope. Scale bar: 20 µm. White arrows highlight gaps between cells. * *p* ≤ 0.05; ** *p* ≤ 0.01; *** *p* ≤ 0.001.

We focused our attention on endothelial permeability, a well-known marker of endothelial function. Figure 5a shows that the cells cultured in low Mg were more permeable to fluorescein isothiocyanate-labeled albumin (FITC-BSA) than the control in 1 mM Mg. Silencing TXNIP restored normal permeability. 

To explore the underlying mechanisms, we analyzed the amounts and localization of two crucial junctional proteins, Ve-cadherin (VECAD), a member of the adherens junction family, and the tight junction protein zonula occludens (ZO)-1, via Western blotting and immunofluorescence. The cells cultured in 0.1 mM Mg were characterized by lower amounts of ZO-1 and VECAD than the controls, and TXNIP silencing restored the physiological levels of the two proteins (Figure 5b). Interestingly, we noticed that the low-Mg-induced increase in TXNIP also affected the localization of both VECAD and ZO-1. Indeed, the cells cultured in 0.1 mM Mg exhibited intercellular gaps whose formation was prevented by silencing TXNIP (Figure 5c,d), in accordance with the results obtained in the permeability assay.

### 2.6. Low-Mg-Induced Increase in TXNIP Affects Ve-Cadherin Stability

**Figure 6:**
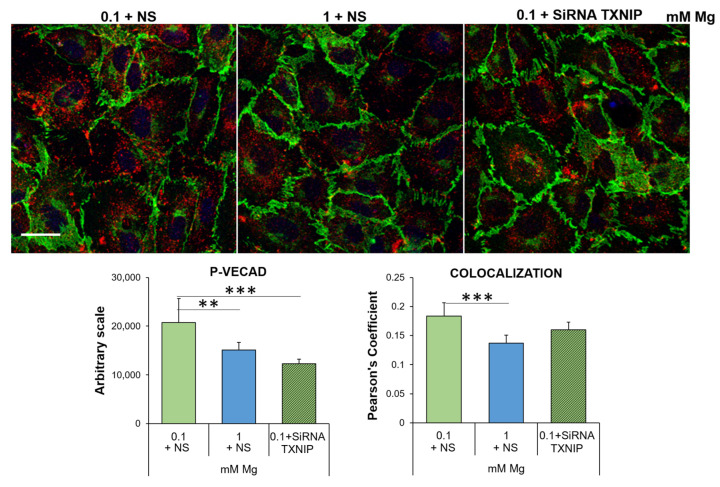
Low-Mg medium induces the phosphorylation of Ve-cadherin in a TXNIP-dependent fashion. Immunofluorescence was performed on HUVEC using antibodies against VECAD and P-VECAD, which were then stained with an Alexa Fluor 488 (green) or 546 (red) secondary antibody, respectively. DAPI (blue) was used to stain the nuclei. Images were acquired using a 40× objective in oil with an SP8 Leica confocal microscope. Scale bar: 20 µm. The quantification of the fluorescence of P-VECAD staining (**left panel**) and of the merging of the green/red fluorescence (**right panel**) is shown below the immunofluorescence. The data are expressed as the mean ± SD. ** *p* ≤ 0.01; *** *p* ≤ 0.001.

As for VECAD, the most important player in regulating the vascular endothelial barrier function [30,31], its levels and linear organization as well as post-translational modifications are crucial to maintain the barrier function. In particular, Ve-cadherin phosphorylation on Tyr658 (P-VECAD) augments permeability [32,33,34]. Through immunofluorescence, we detected higher amounts of P-VECAD in HUVEC cultured in 0.1 mM Mg than in the control, and silencing TXNIP prevented Tyr658 phosphorylation (Figure 6).

## 3. Discussion

Mg has a role in maintaining endothelial integrity and function. Accordingly, in humans, Mg deficiency is linked to an increased risk of coronary heart disease [35], and this finding is explained, at least in part, by the evidence that low levels of Mg promote endothelial dysfunction [3,9,11,36], the initial step in atherogenesis. HUVEC are very sensitive to Mg deprivation. At the transcriptional level, 24 h culture in 0.1 mM Mg modulates the expression of 2728 transcripts [37], mainly involved in inflammatory responses. Accordingly, robust experimental evidence has accumulated regarding the acquisition of a pro-inflammatory, pro-atherogenic and pro-thrombotic phenotype by HUVEC cultured in Mg-deficient media [3,9,38,39,40].

In this work, we first explored the levels of intracellular Mg in HUVEC cultured in 0.1 and 1.0 mM Mg for 24 h and detected no significant differences, a finding that is corroborated by the demonstration that most mammalian cells exposed to Mg deprivation retain their basal intracellular Mg content [41]. It is feasible that the cells adapt to low Mg availability by activating pathways that maintain intracellular Mg concentrations, thus granting basal metabolic activities and membrane integrity, among others. It is the presence of several plasma membrane channels and transporters that guarantees intracellular Mg levels. As we have recently shown [42], HUVEC express the transient receptor potential cation channel subfamily M member (TRPM)7, Magnesium Transporter 1 (MagT1) and solute Carrier family 41 member 1 (SLC41A1). Since HUVEC cultured in 0.1 mM Mg medium upregulate TRPM7 [43], we propose a role for TRPM7 in maintaining intracellular Mg homeostasis in our experimental model. Additionally, free Ca^2+^ does not change, and this is an important issue since Mg has long been considered the physiologic calcium blocker [44].

Mg deficiency promotes oxidative stress. Here, we show that the upregulation of TXNIP is, in part, responsible for the accumulation of ROS, lipid droplets and hyperpermeability in HUVEC exposed to low Mg. Since the intracellular concentration of Mg is comparable in controls and in HUVEC cultured in Mg-deficient medium, a puzzling question remains concerning the mechanisms underlying TXNIP upregulation. Again, we hypothesize a role for TRPM7, whose levels, as just mentioned, increase in response to low extracellular Mg [43]. Indeed, TRPM7 not only transports Mg but is also a kinase that phosphorylates several substrates, thus influencing signal transduction, transcription, translation and protein stability [45]. More studies are necessary to verify if a link exists between TRPM7 upregulation and TXNIP stability. TXNIP participates in the control of the redox balance, by negatively regulating the expression and function of TRX [25,46,47]. This action explains why the upregulation of TXNIP is involved in the pathogenesis of several inflammatory disorders [48]. In vitro TXNIP is increased in hypertension-promoted endothelial dysfunction [49] and in 4-hydroxynonenal-induced endothelial senescence [50]. Therefore, it is not surprising that TXNIP inhibition alleviates H_2_O_2_-induced senescence in HUVEC [51]. Notably, TXNIP upregulation is central in driving the accumulation of lipid droplets in HUVEC exposed to high glucose [29]. Similarly, HUVEC cultured in low-Mg-containing medium for 24 h overproduce triglycerides that are then stored in lipid droplets, an event prevented by silencing TXNIP. Lipid droplets are dynamic organelles that store energy in the form of neutral lipids, but also mitigate stress because they take up toxic lipids and, therefore, protect against toxicity [12,52]. It is feasible that the increase in lipid droplets represents a common adaptive feature of metabolically stressed endothelial cells. Accordingly, lipid droplets are increased in the endothelium lining atheroma and in cultured endothelial cells exposed to high-cholesterol-containing sera [53]. Lipid droplets also curtail mitochondrial fragmentation and ROS production [54]. It is worth noting that no alterations in the mitochondrial content and dynamics were detected in HUVEC in Mg-deficient medium vs. their controls, and we hypothesize that lipid droplets might exert a protective effect. Mitochondria, the power plants of the cell, are the principal intracellular Mg stores, since one third of the total Mg is located in these organelles [55]. Mg regulates mitochondrial function [28], and dysregulated levels of mitochondrial Mg alter cellular energy metabolism [56]. We propose that 24 h culture in Mg-deficient medium does not affect the mitochondrial network and dynamics, probably because of the capacity of HUVEC to maintain cytosolic intracellular Mg homeostasis.

We also demonstrate that low extracellular Mg impairs the endothelial barrier function by altering inter-endothelial junctions, meaning that gaps form between neighboring cells. We show that Mg deficiency disrupts the distribution of VE-cadherin, the main component of endothelial adherens junctions, critically involved in the control of the endothelial barrier [30,31,57]. In HUVEC cultured in low Mg, the opening of the junction is due to the phosphorylation of Y658. Once phosphorylated, VE-cadherin is internalized and ubiquitinated [58]. Since silencing TXNIP restores endothelial permeability as well as the membrane localization of junctional proteins, we hypothesize a role of TXNIP in increasing endothelial permeability by raising ROS levels and, consequently, activating NFkB, thus inducing interleukin 1 [36], a potent inducer of vascular permeability [59]. 

## 4. Materials and Methods

### 4.1. Cell Culture

Human umbilical vein endothelial cells (HUVEC) were obtained from the American Type Culture Collection (ATCC, Manassas, WV, USA) and cultured in medium M199 (Euroclone, Milan, Italy) containing 10% fetal bovine serum (FBS), 1 mM l-Glutamine, 1 mM Sodium Pyruvate, 1 mM Penicillin-Streptomycin, 5 U/mL Heparin and 150 µg/mL Endothelial Cell Growth Factor on 2% gelatin-coated dishes (Euroclone). To analyze the effects of normal (1 mM) and low (0.1 mM) Mg concentrations, HUVEC were cultured in custom-made Mg-free medium (Thermo Fisher Scientific, Waltham, MA, USA) supplemented with Mg sulfate (MgSO_4_) to reach these final concentrations.

### 4.2. Magnesium and Calcium Measurement

For the total intracellular Mg quantification, the DCHQ5 probe (donated by S. Iotti) was used [22]. After 24 h of treatment, HUVEC were trypsinized, collected and counted, and 10,000 cells were used for the analysis. Cells were lysed in phosphate-buffered saline (PBS) and sonicated. Then, the sample was diluted in a 1:1 MOPS (3-(N-morpholino) propanesulfonic acid): MeOH (pH 7.4) solution, and the DCHQ5 probe was added at a final concentration of 15 µM. The fluorescent signal (λexc = 360 nm, λemm = 510 nm) was detected with a Varioskan LUX Multimode Microplate Reader (Thermo Fisher Scientific). 

Free intracellular Mg^2+^ and Ca^2+^ were measured with the fluorescent probes Mag-fura-2AM and Fura-2AM, respectively (Molecular probes, Thermo Fisher Scientific). Cells were seeded on a black 96-well plate (Greiner bio-one, Frickenhausen, Germany), and 24 h after the treatment, cells were incubated for 1 h with the respective probe (Mag-fura-2AM: 2.5 µM; Fura-2AM 10μm). The fluorescent signal was measured (Mag-fura-2AM: λexc = 335 nm, λemm = 510 nm; Fura-2AM: λexc = 340 nm, λemm = 510 nm). The experiment was performed three times in triplicate, and the results are expressed as the mean ± SD.

### 4.3. Western Blot

HUVEC were lysed in 10 mM Tris-HCl (pH 7.4) containing 3 mM MgCl_2_, 10 mM NaCl, 0.1% SDS, 0.1% Triton X-100, 0.5 mM EDTA and protein inhibitors, separated on SDS-PAGE and transferred to nitrocellulose sheets at 400 mA for 2h at 4 °C. Western blot analysis was performed using antibodies against P-HSP27, HSP27, DRP1, pDRP1^Ser616^, pDRP1^Ser637^, OPA1 (Cell Signaling Technology, Danvers, MA, USA), TXNIP, CYP D, ZO-1, VECAD (Thermo Fisher Scientific), HSP70 and Actin (Tebu Bio-Santa Cruz, Magenta, Italy), which were used as a control of loading. After extensive washing, secondary antibodies labeled with horseradish peroxidase (Amersham Pharmacia Biotech Italia, Cologno Monzese, Italy) were used. The Super-Signal chemiluminescence kit (Thermo Fisher Scientific) was used to detect the immunoreactive proteins. All the experiments were performed at least three times, and a representative blot is shown. Densitometry of the bands from three blots was performed with the software ImageLab (Bio-Rad, Hercules, CA, USA), and the results are expressed as the mean ± SD.

### 4.4. ROS Measurement

For the detection of ROS, HUVEC were cultured in a black 96-well plate (Greiner bio-one) and, at the end of the experiments, incubated for 30 min with 10 mM 2′-7′-dichlorofluorescein diacetate (DCFDA) solution (Thermo Fisher Scientific) to detect total intracellular ROS, or with 5 μM MitoSOX Red mitochondrial superoxide indicator (Thermo Fisher Scientific) to detect mitochondrial ROS. The dye emission was monitored (for DCFDA: λexc = 495 nm, λemm = 529 nm; for MitoSOX: λexc = 510 nm, λemm = 580 nm) using a Varioskan LUX Multimode Microplate Reader (Thermo Fisher Scientific). The amount of ROS production was normalized to the cell number counted after trypsinization. The results are the mean of three independent experiments performed in triplicate ± SD.

### 4.5. TXNIP Silencing

TXNIP was silenced using small interfering RNAs (siRNAs). Subconfluent cells were transfected using Lipofectamine RNAiMAX (Invitrogen, Thermo Fisher Scientific) combined with siRNAs targeting TXNIP (20 nmol, 5′-AAGCCGTTAGGATCCTGGCT-3′ (Qiagen, Hilden, Germany)) (0.1 + siRNA TXNIP), while non-silenced samples (0.1 + NS and 1 + NS) were transfected with a scrambled non-silencing (NS) sequence. After 6 h, the siRNA transfection medium was replaced with a culture medium for the respective treatment.

### 4.6. Real-Time PCR

Total RNA was extracted using the PureLink RNAMini Kit (Thermo Fisher Scientific). Single-stranded cDNA was synthesized from 1 mg RNA in a 20 µL final volume using the High-Capacity cDNA Reverse Transcription Kit with RNase inhibitor (Thermo Fisher Scientific), according to the manufacturer’s instructions. Real-time PCR was performed in triplicate using the CFX96 Real-Time PCR Detection system (Bio-Rad) with TaqMan Gene Expression Assay probes for TXNIP (Hs00197750_m1) and GAPDH (Hs99999905_m1) (Life Technologies, Thermo Fisher Scientific). The housekeeping gene GAPDH was used as an internal reference gene. Relative changes in gene expression were analyzed using the 2^−ΔΔCt^ method. The experiment was performed in triplicate three times. Data are expressed as means ± SD.

### 4.7. Immunofluorescence and Confocal Imaging

To stain mitochondria and adhesion molecules, the cells were fixed in PBS containing 4% paraformaldehyde and 2% sucrose (pH 7.6), permeabilized with Triton 0.3% and incubated with antibodies anti-PLIN2 (Abcam, Cambridge, UK), CYP D, VECAD, ZO-1 and P-vecad (Thermo Fisher Scientific) overnight at 4 °C, followed by staining with an Alexa Fluor secondary antibody (Thermo Fisher Scientific). 4′,6-Diamidine-2′-phenylindole dihydrochloride (DAPI, Sigma-Aldrich) was used to stain the nuclei. Finally, the cells were mounted with ProLong™ Gold Antifade Mountant (Thermo Fisher Scientific), and images were acquired using a 40× objective in oil with an SP8 Leica confocal microscope. Alternatively, to label mitochondria for quantification, the cells grown on microscope glasses and treated for 24 h were incubated with medium containing 25 nM of MitoTracker Red CMXRos (Thermo Fisher Scientific) for 15 min at 37 °C, and then the fluorescence was read (λexc = 570 nm, λemm = 599 nm) using a Varioskan LUX Multimode Microplate Reader (Thermo Fisher Scientific). Fluorescence analysis was performed by quantifying the intensity of the staining signal using ImageJ. Colocalization analysis of two proteins was performed using the Jacop plugin of ImageJ to analyze the merging of the two colors. The results are expressed as the mean ± SD.

### 4.8. Triglyceride Quantification

Triglycerides were quantified using a Triglyceride Quantification Kit (Sigma-Aldrich) according to the manufacturer’s instructions. Basically, triglycerides were extracted from cell lysates and broken down into fatty acids and glycerol, which was suddenly oxidized to generate a fluorescent product (λexc = 535 nm, λemm = 590 nm). The fluorescence was measured using a Varioskan LUX Multimode Microplate Reader (Thermo Fisher Scientific). The results were normalized to the cell number, and are presented as the mean of three independent experiments performed in triplicate ± SD.

### 4.9. Bodipy Staining

For the intracellular quantification of neutral lipids, Bodipy staining and quantification were performed (Thermo Fisher Scientific). The cells were seeded on a black 96-well plate, treated for 24 h and then stained following the manufacturer’s instructions. The fluorescence was detected using a Varioskan LUX Multimode Microplate Reader (Thermo Fisher Scientific) (λexc = 493 nm, λemm = 503 nm). The experiment was performed in triplicate three times, and the data are expressed as the mean ± SD.

### 4.10. Transwell Permeability Assay

The Transwell Permeability Assay was performed in a 24-well receiver plate with individual hanging cell culture inserts (Transwell Permeable Supports, 0.4 µm micropores, Euroclone). HUVEC were seeded into the inserts and, when confluent, treated with 0.1 mM Mg + NS, 1 mM Mg + NS or 0.1 + siRNA TXNIP mM Mg for 24 h. At the end of the treatment, 1 mg/mL fluorescein isothiocyanate-labeled albumin (FITC–BSA) (Sigma–Aldrich) was added to the upper part of the transwell, and the extent of permeability was determined by measuring the fluorescence in the lower compartment. The fluorescence was detected using a Varioskan LUX Multimode Microplate Reader (Thermo Fisher Scientific) (λexc = 495 nm, λemm = 519 nm). The experiment was performed in triplicate three times, and the data are expressed as the mean ± SD.

### 4.11. Statistical Analysis

Data are reported as means ± SD. For the experiments with only two experimental conditions (0.1 mM and 1 mM Mg), the data were analyzed using the Student t-test method. For the experiments with three samples (0.1 mM Mg + NS, 1 mM Mg + NS and 0.1 mM Mg + siRNA TXNIP), the data were normally distributed, and they were analyzed using one-way repeated measures ANOVA. The *p*-values derived from the multiple pairwise comparisons were corrected using the Bonferroni method. Statistical significance was defined as a *p*-value of ≤0.05. * *p* ≤ 0.05; ** *p* ≤ 0.01; *** *p* ≤ 0.001.

## 5. Conclusions

Acute and severe Mg deficiency upregulates TXNIP, which contributes to the generation of ROS, with a consequent increase in endothelial permeability and accumulation of triglycerides in lipid droplets. More studies are ongoing to test how HUVEC behave when chronically challenged with very low extracellular Mg concentrations. 

## Data Availability

Data are openly available at Dataverse through the following link: https://dataverse.unimi.it/dataverse/txnip_magnesium, accessed on 28 April 2023.
